# Effect of astaxanthin-rich extract derived from *Paracoccus carotinifaciens* on cognitive function in middle-aged and older individuals

**DOI:** 10.3164/jcbn.17-100

**Published:** 2018-01-27

**Authors:** Masahiro Hayashi, Takashi Ishibashi, Takashi Maoka

**Affiliations:** 1 Biotechnology Development Group, Biotechnology Business Unit, High Performance Materials Company, JXTG Nippon Oil & Energy Corporation, 8 Chidori-cho, Naka-ku, Yokohama-shi, Kanagawa 231-0815, Japan; 2 Biotechnology Business Group, Biotechnology Business Unit, High Performance Materials Company, JXTG Nippon Oil & Energy Corporation, W Building, 1-8-5, Konan, Minato-ku, Tokyo 108-8005, Japan; 3 Research Institute for Production Development, 15 Shimogamohoncho, Sakyo-ku, Kyoto 606-0805, Japan

**Keywords:** astaxanthin, *Paracoccus carotinifaciens*, cognitive function, adonirubin, adonixanthin

## Abstract

This study was conducted to investigate the effect of dietary supplement containing astaxanthin-rich extract derived from *Paracoccus carotinifaciens* (astaxanthin supplement) on cognitive function of subjects aged 45–64 years. Cognitive functions of 28 subjects orally administered 8 mg astaxanthin/day of astaxanthin supplement for 8 weeks (astaxanthin group) and 26 subjects given a placebo (placebo group) were compared by word memory test, verbal fluency test, and Stroop test. The astaxanthin group experienced significantly larger increase in blood astaxanthin level than the placebo group. However, there were no significant intergroup differences in the results of the tests. A subgroup analysis was performed after dividing subjects into the <55 years old and ≥55 years old age groups. The result of “words recalled after 5 minutes” in word memory test in <55 years old subjects showed significant improvement in the astaxanthin group than in the placebo group, which was not found in ≥55 years old subjects. Our results indicate that people aged 45–54 years may experience improved cognitive function after ingesting astaxanthin supplement for 8 weeks. On the basis of the parameters tested, administration of astaxanthin supplement was not associated with any problems related to safety.

## Introduction

Astaxanthin is a carotenoid similar to β-carotene and lycopene and is classified as xanthophylls. Astaxanthin is designated as 3,3'-dihydroxy-β,β-carotene-4,4'-dione in IUPAC nomenclature. It is a red-tinged pigment naturally accumulated in aquatic creatures, such as fish and shrimp, and it is also known to have a powerful antioxidant effect.^(1)^

Astaxanthin is used in animal feed to improve the color of the fish raised in hatcheries, and it has also been sold in human use such as dietary supplements and cosmetics. Several studies have reported on the biological role of astaxanthin, including protection of the retina,^(2)^ amelioration of eye fatigue,^(3)^ enhancement of mitochondria function,^(4)^ promotion of the use of fat as an energy source,^(5)^ increase in motor function,^(6)^ beautification of skin,^(7)^ control of arteriosclerosis,^(8)^ prevention of hyposalivation^(9)^ and inhibitory effect of blood pressure (BP) elevation.^(10)^ Recently, astaxanthin has gained worldwide attention as a multi-functional health ingredient.

In addition to synthetic astaxanthin, natural astaxanthin extracted from various organisms are commonly used. The most commonly used natural form of astaxanthin is derived (or extracted) from *Haematococcus pluvialis* (*H. pluvialis*), a type of green algae.^(11)^ Astaxanthin derived from *H. pluvialis* is used in the majority of functional studies. Astaxanthin is the predominant component of the carotenoids derived from *H. pluvialis*, and only the optically pure (3*S*,3*'S*)-astaxanthin, more than 90% terminal hydroxyl group of which is esterized with fatty acids, has been isolated.^(12)^ In addition, the red yeast *Phaffia rhodozyma* is known to produce astaxanthin.^(13)^ The chemical composition of the astaxanthin that is produced by this yeast is the opposite to the commonly occurring natural astaxanthin (3*S*,3*'S*-configuration) and is designated as the 3*R*,3*'R*-configuration.^(14)^ The terminal ends have hydroxyl groups that are not esterized or modified in any way.^(15)^ *Paracoccus carotinifaciens* (*P. carotinifaciens*) is also known to produce carotenoid compounds that contain astaxanthin.^(16)^ Astaxanthin derived from *P. carotinifaciens* is a “free” form devoid of terminal modification and has a 3*S*,3*'S*-configuration.^(17)^ In addition to astaxanthin, 20–30% of the extract derived from *P. carotinifaciens* consisted of the potent antioxidant adonirubin and adonixanthin.^(18)^ Thus, natural astaxanthin shows a wide variety of characteristics depending on their source.

Healthy aging, which includes improving diet, habits of daily living and life expectancy, has been receiving increasing attention as populations around the world continue to age. In particular, studies on preserving cognitive functions, which declines by aging and dementia, have been conducted. Because omega-3 fatty acids (e.g., docosahexaenoic acid and eicosapentaenoic acid) are known to maintain cognitive function, it is now recommended that people receive 1 g of the fatty acids daily.^(19)^ It is reported that tocotrienol markedly counteracts the decline in learning and memory function in aged rats.^(20)^ Krikorian *et al.*^(21)^ suggest that supplementation of blueberry congaing anthocyanins can confer neurocognitive benefit for older adults. In addition, a study on elderly non-demented people indicated that administering the extract of *Ginkgo biloba* led to a reduction in blood viscosity, improvement of cerebral perfusion in specific areas and global cognitive functioning.^(22)^ Moreover, it has been shown that the use of an astaxanthin-rich extract from *H. pluvialis* leads to an improvement in cognitive function.^(23)^ Therefore, there is considerable evidence supporting the effectiveness of dietary supplements on improving cognitive function, and it is likely that such supplements may be useful for preventing dementia.

In the present study, we compared the improvements in cognitive function by word memory test, verbal fluency test and Stroop test between middle-aged and old-aged subjects who were administered dietary supplement containing astaxanthin-rich extract derived from *P. carotinifaciens* and subjects who received a placebo. Unlike the extract derived from *H. pluvialis*, the astaxanthin-rich extract derived from *P. carotinifaciens* has not been subjected to extensive clinical study. The astaxanthin-rich extract derived from *P. carotinifaciens* has different features such as free-form astaxanthin, adonirubin and adonixanthin, compared with that derived from *H. pluvialis*. Therefore, the present study suggests not only additional evidence of astaxanthin supplement but also efficacy of astaxanthin supplement differently derived, which will be helpful to develop dietary supplements for cognitive function and promote carotenoid research.

## Materials and Methods

### Study design

This study was a randomized, double-blind, placebo-controlled, parallel inter-group comparison.

### Subjects

**Fig. 1:**
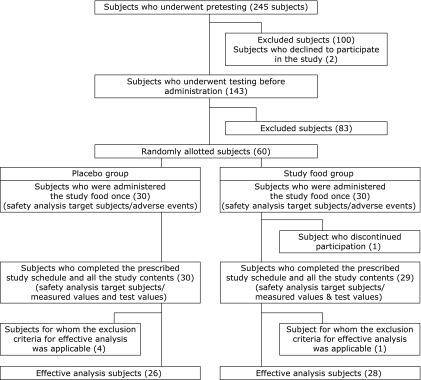
Classifications and details of the subjects.

Classification and details regarding subjects who participated in this study are shown in Fig. 1. The placebo group was designated as group P and the astaxanthin group was designated as group A. A total of 245 healthy subjects aged 45–64 years old were initially identified as candidates for this study. On the basis of results of pre-study tests [physical tests and the Hasegawa Dementia Scale-Revised (HDS-R)] and pre-administration tests (medical interview and cognitive function tests), 60 subjects (group P: *n* = 30; group A: *n* = 30) were determined to be appropriate for participation as subjects and were enrolled in this study.

Of the 60 total subjects, one subject (ID 4245: group A) developed contact dermatitis (arms, left foot and face) on the 9th day of administration and discontinued the study to undergo treatment. The remaining 59 subjects completed the prescribed study schedule and performed all study tests. In addition, four subjects in group P and one in group A were excluded for per-protocol analysis because one of them was unconfirmed to ingest the test supplement, and the others changed their drinking habit, sport habit or working arrangement during this study. Thus, only the remaining 54 subjects (group P: *n* = 26; group A: *n* = 28) were available for efficacy analysis. Regarding safety, 60 adverse events (30 in group P, 30 in group A) were observed in subjects who were administered the supplement preparations even once. Data of all 59 of the original 60 subjects (group P: *n* = 30; group A: *n* = 29) who completed the study schedule and all study procedures were used in our analysis of safety. The administration rate of the supplement was between 94.6 and 106.7% for all 59 subjects.

### Clinical registration

This study has been registered at University hospital Medical Information Network (https://upload.umin. ac.jp/cgi-open-bin/ctr_e/ctr_view.cgi?recptno=R000025682) on 15th May 2016 as “Efficacy study of oral astaxanthin to improve brain function for the middle aged and elderly”, Identification No. UMIN000022299.

### Supplements

We prepared an astaxanthin-rich extract from *P. carotinifaciens* using the method described by Katsumata *et al.*^(17)^ We then added arabic gum, maltodextrin, *dl*-α-tocopherol, medium-chain triglycerides, and l-ascorbic acid to the astaxanthin-rich extract, mixed the ingredients and dissolved them in water. After emulsification, we spray-dried the mixture to obtain 1% astaxanthin powder which also contains 0.14% adonirubin and 0.05% adonixanthin. We produced a jelly containing 2 mg of astaxanthin, which contains 1% astaxanthin powder, pH adjuster, sweetener, gelling agent, flavor (mixed berry) and water, and then it was administered to subjects in group A as astaxanthin supplement (Table 1). As a substitute, subjects in group P were given jelly containing artificial red coloring (Food Red No. 2, Amaranth) that was prepared the same method except 1% astaxanthin powder as the supplement provided to group A. The astaxanthin-rich extract from *P. carotinifaciens* and 1% astaxanthin powder were examined in terms of safety by Food and Drug Administration in United States, and have been filed as New Dietary Ingredients.

In previous studies with astaxanthin, 6 mg of astaxanthin per day and 12 mg of astaxanthin per day were administered to subjects for 12 weeks, and their cognitive function and erythrocyte antioxidant status were improved in the both groups.^(23,24)^ Because the administration period of this study was 8 weeks which was shorter than those of the previous studies, the astaxanthin amount per day of administration was decided to 8 mg of astaxanthin per day in this study. All subjects were requested to ingest two jellies of their respective supplement preparations after breakfast and again after dinner for 8 weeks to be administered four jellies (8 mg of astaxanthin) per day or placebo.

### Institutional review board

This study was conducted according to the Declaration of Helsinki (October 2013 revision). The Ethical Guidelines for Biomedical Research Involving Human Subjects (Ministry of Education, Culture, Sports, Science and Technology and the Ministry of Health, Labour and Welfare, December 22, 2014) and the Ethical Guidance for Biomedical Research Involving Human Subjects [Ministry of Education, Culture, Sports, Science and Technology, and the Ministry of Health, Labour and Welfare, February 9, 2015 (March 31, 2015 partial revision)] were followed.

### Physical measurements

We measured the subjects’ height and weight, calculated their body mass index (BMI) and conducted the following tests: systolic and diastolic BP levels, heart rate, hematologic test, biological examination of blood and urine test (protein, sugar and occult blood).

### Analysis of astaxanthin, adoniurbin, and adonixanthin concentration in human serum

**Fig. 2:**
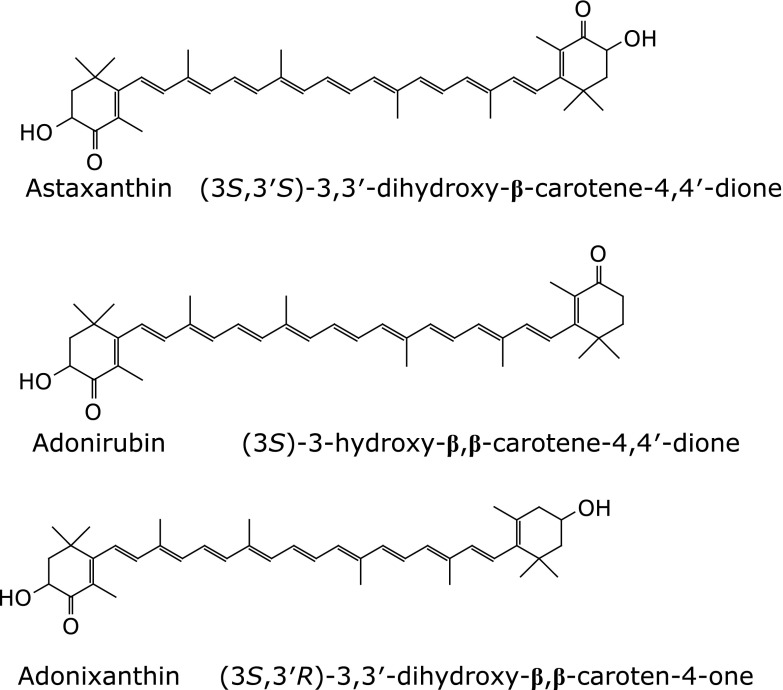
Structural formula of astaxanthin, adonirubin and adonixanthin.

The astaxanthin, adonirubin and adonixanthin (Fig. 2) concentration in human serum were analyzed by the method described by Fukuda *et al.*^(25)^ A 1 ml of water and a 1 ml of ethanol, containing 0.85 µg of β-apo-8'-carotenal (internal standard) and 50 µg of 2,6-dibuthylhydroxytoluene, was added to 300 µl of serum. Then, 5 ml of hexane was added to the mixture and was shaken for 20 min. Then the hexane layer was taken in another test tube after centrifugation [4°C, 3,000 rpm (1,464 × *g*), 10 min] and was dried over under nitrogen gas flow. The residue was dissolved in 100 µl of chloroform/ethanol and subjected to UPLC-MS/MS analysis using Cadenza CD-C18 column with mixture of 85% acetonitrile in 2 mM ammonium acetate and acetonitrile/methanol/tetrahydrofuran (60:38:2) in 2 mM ammonium acetate as solvent. Detection and quantification were performed by selective reaction monitoring (SRM) of protonated molecule (MH+) of astaxanthin, adonirubin, and adonixanthin.

### Cognitive function tests

#### Word memory test

The word memory test assesses the capacity of the subjects’ immediate and short-term memory as well as retention and ability to repeat words. We performed a test based on the references.^(26,27)^ After reading seven words aloud one at a time to the subjects, they were asked to repeat the words. Immediately after they had repeated the seventh word, they were asked to recall all of the words (number of immediate-recall words). The subjects were given hints consisting of the initial sounds and categories of the words when they could not spontaneously recall. Words that they were then able to recall were recorded as the number of cued-recall words. In addition, subjects were given interference tasks [(2) Verbal fluency test] and were then asked to recall the words 5 min after the immediate-recall test (number of words recalled after 5 min). Once again, the subjects were given hints consisting of the initial sounds and categories of the words when they were unable to recall. The words which they were then able to recall were recorded as the number of cued-recall words. We then assessed the number of immediate-recall words, immediate-recall words + cued-recall words, recalled words after 5 min, and recalled words after 5 min + cued-recall words. Increases in the measured values indicate a memorization power improving action.

#### Verbal fluency test

The verbal fluency assessed the subjects’ memory-recall ability and was based on the reference.^(28)^ In the “vegetable-verbal fluency test,” the subjects were instructed to “say the names of the vegetables to the greatest extent possible for 1 min.” After the first list was read, the number of words that could be recalled in 60 s was recorded and did not include duplicates. The same procedure was used for lists of words beginning with the words for “a” and lists of animal names. These tests were performed before administration and at the 4th and the 8th week after administration in differing order. Numbers of words the subjects were able to correctly list in each test were assessed. Increases in the number of correct answers indicate a cognitive function improving.

#### Stroop test

The Stroop test assesses the brain’s ability to distinguish and process two different types of data (verbal and color information) that enter the brain simultaneously. We followed the testing procedure of the New Stroop Test II^(29,30)^ which consists of four steps (steps 1, 2, 3 and 4), each considered as separate sub-tests. In step 1, the subjects were asked to mark a check-box of the color of the ink indicated by the word. In step 2, the subjects were asked to mark a check-box of the color of the ink indicated by the word which did not match the color of ink. In step 3, the subjects were asked to select the word that corresponded to the color of the ink and mark the check box. In step 4, the subjects were asked to select the word that corresponded to the color of the ink used to write the word which did not match the color of the ink and mark the check-box. As the steps advance, the level of difficulty increases. The number of correct answers at each step was assessed. Increases in the measured values for the number of correct answers indicate improvement of brain information processing ability.

### Sample size

We estimated sample size based on Word memory test (logical memory immediate retrieval) from data published by Santos *et al.*^(22)^ It was assumed that the mean difference would be 2.0, with a SD of 2.5. To detect this difference, with a power of 80% and a significance level of 5% and taking into account that 10% of the patients will be lost to follow-up, it was calculated that thirty patients would be needed to be studied in each group.

### Statistical analysis

For the subject background factors, a chi-square test was used to compare sex (male/female) between group A and P, and 2-sample *t* test was also used to compare the others between both groups. For the serum concentrations of carotenoid and cognitive function tests, 1-sample *t* test was used to compare changes between pre-administration baseline values to those at 4 weeks and 8 weeks after administration in groups A and P. Two-sample *t* test was also used to compare the amount of change between both groups in the serum concentrations of carotenoid. For cognitive function tests, we used two-way-repeated measures of ANOVA to compare the amount of change between both groups. Numerical values were expressed as the mean and SD, and the standard of significance for the tests was set at 5% on both sides. All statistical analysis was performed using IBM SPSS Statistics 23.

## Results

### Background factors of subjects

The background factors of the 54 subjects who underwent effective analysis are listed in Table 2. The mean age of subjects in group P was 54.4 ± 6.0 years old, and there were a total of 13 men and 13 women. The mean age of the subjects in group A was 56.0 ± 5.2 years old, and there were 12 men and 16 women. HDS-R scores were 28.6 ± 1.2 and 28.5 ± 2.2 points in groups P and A, respectively, both of which were within the normal range. No intergroup differences were observed in data pertaining to the subjects’ age, sex, HDS-R score, physical measurement, or physical examination.

### Blood carotenoid levels

Table 3 presents changes in the measured values and the amount of change compared with the baseline for blood astaxanthin, adonirubin and adonixanthin levels. In group A, the values at 8 weeks after administration for all items were associated with significantly greater increases than group P (group P vs group A; astaxanthin: 0.000 ± 0.000 vs 0.173 ± 0.058 µg/ml, adonirubin: 0.000 ± 0.000 vs 0.042 ± 0.014 µg/ml and adonixanthin: 0.000 ± 0.000 vs 0.016 ± 0.006 µg/ml). In comparison with the baseline, though the values at 8 weeks after administration did not increase in group P, all the values at 8 weeks after administration were significantly elevated in group A.

### Cognitive function tests

Table 4 presents changes in the measured values and the amount of change compared with the baseline for all the items of Word memory test, Verbal fluency test and Stroop tests.

#### Word memory test

There were no significant intergroup differences regarding the amount of change after administration for any of the items. In comparison with the baseline, the number of recalled words after 5 min was significantly increased 8 weeks after administration in group P. Moreover, the number of immediate-recall words 4 weeks after administration and recalled words after 5 min + cued-recall words 8 weeks after administration were significantly increased in group A compared with the baseline.

#### Verbal fluency test

There were no observed significant intergroup differences in the amount of change after administration for any of the items. In comparison with the baseline, words that begin with “a” 8 weeks after administration and animal words at both 4 and 8 weeks after administration exhibited significant increases in group P, and all items at 8 weeks after administration were significantly elevated in group A.

#### Stroop test

There were no significant intergroup differences in the amount of change after administration for any of the items. Compared with the baseline, there were significant increases at 8 weeks after administration for step 1 in group P and at 4 and 8 weeks after administration for group A. In addition, both groups were associated with significant increases at 4 and 8 weeks after administration for steps 2 and 3. Group A showed significant increases at 8 weeks after administration for step 4.

### Additional analysis by the age group

Salthouse^(31)^ reported the existence of cross-sectional age-related declines for many cognitive variables prior to age 60. Therefore, as an exploratory effective analysis, we divided the subjects into two age groups: (1) <55 (45–54) years old and (2) ≥55 (55–64) years old. We then performed an additional analysis for their cognitive functions by age group. The background factors of the subjects by age group are shown in Table 5A and B. Intergroup differences between all subjects aged <55 years old and ≥55 years old in Table 5A were observed in data pertaining to the subjects’ age, weight, BMI, systolic pressure and diastolic pressure (<55 years old vs ≥55 years old; age: 50.0 ± 2.9 years vs 59.7 ± 2.8 years, weight: 56.63 ± 9.90 kg vs 63.88 ± 13.91 kg, BMI: 21.59 ± 2.58 kg/m^2^ vs 23.40 ± 3.28 kg/m^2^, systolic pressure: 113.0 ± 15.1 mmHg vs 133.0 ± 15.2 mmHg and diastolic pressure: 68.4 ± 11.3 mmHg vs 79.4 ± 10.9 mmHg). Heart rate of the subjects aged <55 years old was lower than that of the subjects aged ≥55 years old (*p*<0.1). In addition, the HDS-score of the subjects aged <55 years old was larger than that of the subjects aged ≥55 years old (*p*<0.1). No intergroup differences between group P and group A divided by age 55 were observed in data pertaining to the subjects’ age, sex, HDS-R score, physical measurement, or physical examination (Table 5B).

Changes in the measured values and the amount of change for all items of the subjects aged <55 years old and aged ≥55 years old in comparison to the baseline in the cognitive function tests are presented in Table 6A and B respectively. In subjects aged <55 years old, the group A indicated statistically significant improvement in the number of word recalled after 5 min of Word memory test, compared to the placebo group (group P vs group A; at 4 weeks: –0.4 ± 0.8 vs 1.2 ± 1.5 points, at 8 weeks: 0.9 ± 1.4 vs 1.2 ± 1.3 points). In addition, the number of immediate-recall words + number of cued-recall words and the number words recalled after 5 min + number of cued-recall words in group A were larger than those of group P (*p*<0.1). An investigation into the amount of change for all items in Word memory test among the subjects aged ≥55 years old showed no significant intergroup differences. Also, there were no significant intergroup differences in the amount of change after administration for any of the items in Verbal fluency test and Stroop test for both age groups.

### Safety assessment

We believe that the cause of the rash (i.e., redness and slight itchiness) that was observed on subject ID 4245 (group A) who discontinued the study was related to the subject trimming garden trees on the day before the onset of the rash, resulting in a case of contact dermatitis. The principal investigator (a physician) determined that the rash was “unrelated” to the supplement.

Subject ID 4161 (group P) developed mild acne vulgaris on nine occasions between the 17th and 48th day after administration. Although the cause could not be identified, the intermittent appearance of acne disappeared without the use of medication and it did not reappear after the 48th day after administration. On the basis of these observations, the principal investigator determined that the condition was “likely unrelated” to the supplement.

Investigation of other adverse events indicated that all were “unrelated” to the supplement. Therefore, we concluded that the supplement caused no adverse events (side effects). Results of our investigation regarding changes in numerical values for physical measurements (including blood and urine tests) in all groups indicated that although there were minor changes, none were determined to be clinically significant.

## Discussion

Astaxanthin is a powerful antioxidant that can penetrate the blood-brain barrier, and it is thus often used in research related to cognitive function. Nakagawa *et al.*^(24)^ reported that the astaxanthin administration led to a reduction in phospholipid peroxidation in red blood cells. Because patients with dementia are known to have increased phospholipid peroxidation in their red blood cells, ingesting astaxanthin is considered to be putative preventative effect against dementia. In addition, a study utilizing a model of Parkinson’s disease using 6-hydroxydopamine indicated that adding astaxanthin suppressed neuronal apoptosis.^(32)^ Furthermore, astaxanthin administration has been shown to decrease amyloid-β levels in red blood cells, which is considered to decrease the ability of red blood cells to supply the brain with oxygen;^(33)^ thus, astaxanthin administration might be useful for a treatment of Alzheimer’s disease. It has also been reported that astaxanthin suppresses the extent of oxidative stress in cases of cerebral ischemia and has a neuro-protective effect.^(34)^ Moreover, it was reported that astaxanthin administration in mice has a concentration-dependent effect of promoting neurogenesis in the hippocampus, which is related to learning and memory, suggesting that astaxanthin contributes to memory improvement.^(35)^ Thus, several studies have verified the fact that astaxanthin is effective in improving the brain and neuronal function and is prophylactic against cerebral damage.

The age of the subjects in the present study was 45–64 years old. Because age may affect how astaxanthin administration influences cognitive function, all 54 participants were not only subjected to an effective analysis as a whole but also a subgroup analysis after dividing them into <55 years old and ≥55 years old groups. Cognitive functions were assessed using Word memory test, Verbal fluency test, and Stroop test.

The results of serum carotenoid level measurements (astaxanthin, adonirubin and adonixanthin) in the subjects of group A indicated that astaxanthin, adonirubin and adonixanthin were absorbed into blood after administration of astaxanthin supplement. Ohi *et al.*^(36)^ reported that maximum plasma astaxanthin concentration of subjects taking a soft gel capsule as single dose containing astaxanthin of 9 mg derived from *H. pluvialis* was 0.077 ± 0.066 µg/ml, which was half lower than the concentration in group A of the present study (0.173 ± 0.058 µg/ml). It could be caused by the difference between single administration and repeated administration. Also, the carotenoid ratio in serum of subjects were astaxanthin:adonirubin:adonixanthin = 1:0.24:0.09 and that of astaxanthin supplement were astaxanthin:adonirubin: adonixanthin = 1:0.14:0.05. Consequently, the bioavailability of adonirubin and adonixanthin were superior to astaxanthin. Absorption process of carotenoids requires release of carotenoids from food matrix, diffusion in lipid emulsion, solubilization by pancreatic lipases and bile salts, formation of mixed micelles, movement across the microvilli, uptake of carotenoids by intestinal mucosal cells, incorporation into chylomicrons and circulation to lymphatic.^(37)^ The difference of the molecular structures between astaxanthin, adonirubin and adonixanthin might affect the absorption process, which resulted in the shift of the ratio. We think the serum carotenoid levels in group A were maintained during this study.

We did not detect any significant intergroup differences in the amount of change in any outcome measure for the cognitive function tests among the 54 subjects, while group A showed significantly improvements from their baseline in more items of all the tests compared with group P. An analysis according to age group indicated that subjects aged <55 years old in group A had significantly larger increase in the number of recalled words after 5 min in the Word memory test than that in group P. These finding indicate a certain improvement in group A. It is likely that this result is related to the neurogenesis effect of astaxanthin in the hippocampus that was reported by Yook *et al.*^(35)^ In addition, this may be reasonable because other substances which have antioxidant ability such as omega-3 fatty acids,^(19)^ tocotrienol^(20)^ and anthocyanins^(21)^ are reported to be effective on cognitive function. The weight, BMI, systolic pressure and diastolic pressure of the subjects aged ≥55 years old were significantly higher than those of the subjects aged <55 years old. It was reported that high BP in middle age is a risk factor for late-life cognitive decline and dementia.^(38)^ Hence, it might be difficult for people aged ≥55 years old to improve their cognitive function with astaxanthin supplement for a short period of time like 8 weeks due to the influence of higher BP. Currently, it remains unclear whether astaxanthin supplement administration by human for 4–8 weeks has an effect of suppressing oxidation in the brain and accumulation of amyloid-β; however, astaxanthin supplement may be effective for preventing dementia if ingested from a young age. Further investigation into the molecular and cellular mechanisms of astaxanthin that lead to these improvements in cognitive functions is required.

Previous clinical studies regarding the effect of astaxanthin administration on cognitive functions include the study by Katagiri *et al.*^(23)^ involving astaxanthin derived from *H. pluvialis*. The results of that study suggested that astaxanthin administration improved cognitive function, despite the lack of significant differences between astaxanthin and placebo groups. Although the method of assessing cognitive functions used in the present study differ from that employed by Katagiri *et al.*,^(23)^ because similar doses (the study of Katagiri *et al.*^(23)^ vs the present study; asthaxanthin doses: 0, 6 and 12 mg/day vs 0 and 8 mg/day) and subjects (the subjects were aged 45–64 years old in both studies) were used, it is likely that the presence of statistically significant differences is related to the source of astaxanthin. Besides astaxanthin, adonirubin and adonixanthin are found between 20 and 30% in carotenoid derived from *P. carotinifaciens*, and can efficiently quench singlet oxygen.^(18)^ Inoue *et al.*^(39)^ reported that adonirubin and adonixanthin suppressed reactive oxygen species production and protected cells against light-induced damage as astaxanthin, and adonixanthin protected against light-induced cell damage through not only an anti-oxidative response but also through Nrf2 activation. In addition, it was reported that human neurodegenerative diseases have been associated with inflammation and dysregulation of the Nrf2 system.^(40)^ Therefore, adonirubin and adonixanthin exhibit a multiplier effect with astaxanthin in terms of the antioxidant properties and biological activations like Nrf2, which may explain why a greater improvement in cognitive function was observed in the present study. Further investigation into the effect of different derivations is required.

Regarding safety, an investigation of adverse events indicated that subject ID 4161 (group P) developed acne vulgaris; however, the principal investigator determined this to be “likely unrelated” to the supplement. All other adverse events were determined to be “unrelated” to the supplement. There were no abnormalities in the physical measurements or other tests performed. Although there were some significant differences observed in several items compared with the baseline, under the conditions of the present study, we concluded that astaxanthin supplement was safe for administration. This conclusion is consistent with the findings of Katsumata *et al.*,^(17)^ who reported that repeated administration of astaxanthin derived from *P. carotinifaciens* by rats over 13 weeks at a dose of 1,000 mg/day was not associated with negative effects.

## Authors’ Contributions

MH and TI designed the study, and wrote the initial draft of the manuscript. TM contributed to interpretation of data, and assisted in the preparation of the manuscript.

## Figures and Tables

**Table 1:** Comparison of Supplements per day

Item	Astaxanthin supplement		Placebo	
Form	10 g of jelly × 4 pieces		10 g of jelly × 4 pieces	
Ingredients (mg)	1% Astaxanthin powder	800	Food coloring	2
	pH adjuster	896	pH adjuster	896
	Sweetener	4,092	Sweetener	4,092
	Gelling agent	630	Gelling agent	630
	Flavor	200	Flavor	200
	Water	33,382	Water	34,180
Astaxanthin content (mg)	8 mg		—	
Energy (kJ)	92		92	
Protein (g)	0.04		0.04	
Fat (g)	0.16		0.15	
Carbohydrate (g)	6.04		6.03	
Sodium (mg)	108		108	

**Table 2:** Subject background factors

Item	Group P (*n* = 26)			Group A (*n* = 28)		*p*
	Mean	SD		Mean	SD	
Age (years)	54.4	6.0		56	5.2	0.307
Sex (male/female)	Males: 13/Females: 13			Males: 12/Females: 16		0.785
Height (cm)	163.85	9.04		162.38	9.2	0.557
Weight (kg)	59.67	13.11		61.31	12.38	0.638
BMI (kg/m^2^)	22	3.09		23.09	3.04	0.197
Systolic BP (mmHg)	123.3	17.9		124.1	18.5	0.878
Diastolic BP (mmHg)	74.5	13.1		74.1	11.8	0.900
Heart rate (bpm)	71.2	8.9		68.3	10.1	0.280
HDS-R score (points)	28.6	1.2		28.5	2.2	0.816

Inter-group comparisons with group P (sex: chi-square test; items other than sex: 2-sample *t* test).

BP, blood pressure; bpm, beats per minutes; HDS-R, physical tests and the Hasegawa Dementia Scale-Revised.

**Table 3:** Serum concentration of carotenoid

Item	Numerical item	Group	Baseline			Week 8		*p* (Inter-group)
			Mean	SD		Mean	SD	
Astaxanthin (µg/ml)	Measured value	P	0.000	0.000		0.000	0.000	
		A	0.000	0.000		0.173*	0.058	

	Amount of change	P				0.000	0.000	0.000
		A				0.173	0.058	

Adonirubin (µg/ml)	Measured value	P	0.000	0.000		0.000	0.000	
		A	0.000	0.000		0.042*	0.014	

	Amount of change	P				0.000	0.000	0.000
		A				0.042	0.014	

Adonixanthin (µg/ml)	Measured value	P	0.000	0.000		0.000	0.000	
		A	0.000	0.000		0.016*	0.006	

	Amount of change	P				0.000	0.000	0.000
		A				0.016	0.006	

Group P: *n* = 26, group A: *n* = 28. Intra-group comparison at baseline *p<0.01 (1-sample *t* test). Inter-group comparison with group P (2-sample *t* test).

**Table 4:** Number of answers in cognitive tests

Item		Numerical item	Group	Baseline			Week 4			Week 8		*p* (Inter-group)
				Mean	SD		Mean	SD		Mean	SD	
Word memory test	Immediate recall	Measured value	P	4.6	1.1		4.7	1.0		4.9	1.0	
			A	4.6	0.8		5.0*	0.9		5.1	1.0	

		Amount of change	P				0.0	1.5		0.3	1.7	0.600
			A				0.4	1.0		0.5	1.3	

	Immediate recall + cued recall	Measured value	P	5.8	0.9		5.6	1.0		6.1	0.9	
			A	5.8	0.9		6.1	0.9		6.3	0.7	

		Amount of change	P				–0.2	1.2		0.3	1.3	0.317
			A				0.3	1.2		0.4	1.3	

	Recall after 5 min	Measured value	P	4.0	1.5		4.0	1.6		4.8*	1.5	
			A	4.5	1.3		4.8	1.3		4.9	1.4	

		Amount of change	P				0.0	2.1		0.8	1.7	0.389
			A				0.2	1.5		0.3	1.8	

	Recall after 5 min + cued recall	Measured value	P	5	1.2		4.8	1.3		5.5	1.3	
			A	5.3	1		5.3	1.3		5.9*	0.8	

		Amount of change	P				–0.2	1.7		0.5	1.7	0.876
			A				0.0	1.5		0.6	1.3	

Verbal fluency test	Names of vegetables	Measured value	P	16.6	3.4		16.2	4.1		17.2	3.5	
			A	16	3.1		16.8	3.3		17.4**	3.8	

		Amount of change	P				–0.4	3.1		0.6	3.1	0.348
			A				0.7	2.9		1.4	2.5	

	Words that begin with ”a”	Measured value	P	13.6	3.7		14.1	4.4		15.0*	4.5	
			A	13.7	3.8		13.9	4.2		16.3**	3.6	

		Amount of change	P				0.5	2.8		1.5	3.5	0.361
			A				0.2	4		2.5	3.9	

	Animal words	Measured value	P	18.3	4.8		20.0*	4.8		20.5**	5.1	
			A	17.9	4.4		19.1	4.6		20.1**	4.5	

		Amount of change	P				1.7	4		2.2	3.8	
			A				1.3	4		2.3	2.9	0.865

Stroop test	Step 1	Measured value	P	56.8	6.1		58.1	6.5		61.2**	7.6	
			A	58	6.3		60.7**	6.9		63.6**	6.5	

		Amount of change	P				1.3	5.3		4.5	5.4	0.545
			A				2.7	4.9		5.6	4.2	

	Step 2	Measured value	P	48.2	5.7		50.2*	5.7		52.4**	6.9	
			A	49.3	5.7		51.5*	5.5		53.5**	5.1	

		Amount of change	P				2.0	3.9		4.2	5.3	0.977
			A				2.2	4.6		4.2	4.3	

	Step 3	Measured value	P	38.6	3.9		40.0*	4.6		41.6**	5	
			A	39.6	3.6		41.5**	4.4		42.8**	4.7	

		Amount of change	P				1.4	2.9		3.0	3.0	0.824
			A				1.9	3.5		3.2	3.7	

	Step 4	Measured value	P	33.6	3.8		35.2	5.6		35.6	7.8	
			A	33.5	4.3		35.1	4.4		36.3**	5.3	

		Amount of change	P				1.6	5.5		2.0	6.9	0.863
			A				1.5	5.1		2.8	4.9	

Group P: *n* = 26, group A: *n* = 28. Intra-group comparison at baseline *p<0.05 and **p<0.01 (1-sample *t* test). Inter-group comparison with group P (two-way-repeated measures of ANOVA).

**Table 5A:** Subject background factors of all subjects aged <55 years old and ≥55 years

Item	<55 years (*n* = 25)			≥55 years (*n* = 29)		*p*
	Mean	SD		Mean	SD	
Age (years)	50	2.9		59.7	2.8	0.000
Sex (males/females)	Males: 9/Females: 16			Males: 16/Females: 13		0.182
Height (cm)	161.55	7.9		164.41	9.91	0.252
Weight (kg)	56.63	9.9		63.88	13.91	0.034
BMI (kg/m^2^)	21.59	2.58		23.4	3.28	0.03
Systolic pressure (mmHg)	113	15.1		133	15.2	0.000
Diastolic pressure (mmHg)	68.4	11.3		79.4	10.9	0.001
Heart rate (bpm)	67	7.5		72	10.6	0.058
HDS-R score (points)	29	0.9		28.2	2.3	0.092

Inter-group comparisons with group P (sex: chi-square test; items other than sex: 2-sample *t* test).

bpm, beats per minutes; HDS-R, physical tests and the Hasegawa Dementia Scale-Revised.

**Table 5B:** Subject background factors of subjects in group P and A divided by age 55 years

Item	<55 years						≥55 years				
	Group P (*n* = 14)		Group A (*n* = 11)		*p*		Group P (*n* = 12)		Group A (*n* = 17)		*p*
	Mean	SD	Mean	SD			Mean	SD	Mean	SD	
Age (years)	49.7	3.1	50.5	2.7	0.537		59.9	3.1	59.6	2.6	0.759
Sex (males/females)	Males: 6/Females: 8		Males: 3/Females: 8		0.677		Males: 7/Females: 5		Males: 9/Females: 8		1.000
Height (cm)	161.98	9.06	161.01	6.52	0.768		166.03	8.9	163.26	10.69	0.470
Weight (kg)	57.64	12.12	55.35	6.39	0.577		62.04	14.34	65.17	13.88	0.560
BMI (kg/m^2^)	21.8	3.09	21.32	1.87	0.657		22.23	3.22	24.23	3.15	0.106
Systolic pressure (mmHg)	114	16	111.6	14.5	0.706		134.2	13.6	132.1	16.5	0.727
Diastolic pressure (mmHg)	68.4	11.5	68.4	11.6	0.999		81.7	11.4	77.8	10.6	0.352
Heart rate (bpm)	68.1	6.2	65.6	9.1	0.421		74.7	10.4	70.1	10.6	0.255
HDS-R score (points)	28.9	1.0	29.2	0.8	0.389		28.3	1.4	28.1	2.7	0.753

Inter-group comparisons with group P (sex: chi-square test; items other than sex: 2-sample *t* test).

bpm, beats per minutes; HDS-R, physical tests and the Hasegawa Dementia Scale-Revised.

**Table 6A:** Number of answers of subjects aged <55 years in cognitive tests

Item		Numerical item	Group	Baseline			Week 4			Week 8		*p* (Inter-group)
				Mean	SD		Mean	SD		Mean	SD	
Word memory test	Immediate recall	Measured value	P	4.7	1.4		4.6	1.1		5.1	1.1	
			A	4.6	0.7		5.0	0.9		5.5*	1.1	

		Amount of change	P				–0.1	1.8		0.4	2.1	0.650
			A				0.4	1.0		0.9	1.1	

	Immediate recall + cued recall	Measured value	P	5.9	1.0		5.7	0.8		6.3	1.0	
			A	5.6	0.8		6.5*	0.7		6.5*	0.7	

		Amount of change	P				–0.1	1.2		0.4	1.4	0.085
			A				0.9	1.1		0.9	1.1	

	Recall after 5 min	Measured value	P	4.5	1.3		4.1	1.4		5.4*	1.2	
			A	4.4	1.4		5.5*	0.9		5.5*	1.1	

		Amount of change	P				–0.4	1.8		0.9	1.4	0.027
			A				1.2	1.5		1.2	1.3	

	Recall after 5 min + cued recall	Measured value	P	5.4	1.2		5.0	1.0		6.0	1.2	
			A	5.1	0.9		6.0	1.1		6.2**	0.8	

		Amount of change	P				–0.4	1.6		0.6	1.5	0.074
			A				0.9	1.4		1.1	1.0	

Verbal fluency test	Names of vegetables	Measured value	P	17.4	3.5		17.1	3.0		18	3.4	
			A	15.9	2.2		17.7*	2.8		17.6**	2.8	

		Amount of change	P				–0.2	2.8		0.6	3.0	0.11
			A				1.8	2.1		1.7	1.7	

	Words that begin with “a”	Measured value	P	13.7	3.7		13.2	3.6		15.6	5.4	
			A	14.1	3.2		14.4	4.8		17.5**	3.2	

		Amount of change	P				–0.5	3.0		1.9	3.9	0.607
			A				0.3	4.3		3.4	2.9	

	Animal words	Measured value	P	19.6	3.6		20.5*	3.3		21.1*	3.7	
			A	19.5	4.5		20.4	4.9		22.5*	3.8	

		Amount of change	P				0.9	1.4		1.6	2.2	0.437
			A				0.8	4.8		2.9	3.3	

Stroop test	Step 1	Measured value	P	55.6	6.0		58.6*	6.0		62.7**	6.5	
			A	60.6	5.4		62.6	5.8		67.6**	4.8	

		Amount of change	P				2.9	4.7		7.1	3.5	0.836
			A				2.0	5.1		7.0	4.2	

	Step 2	Measured value	P	48.6	5.6		50.7*	6.7		53.9**	5.8	
			A	51	4.6		52	4.3		54.6**	4.9	

		Amount of change	P				2.1	3.3		5.4	3.3	0.510
			A				1.0	3.9		3.6	2.9	

	Step 3	Measured value	P	38.9	4.4		41.0**	5.2		42.0**	5.8	
			A	40.7	3.0		41.5	4.7		44.3*	5.0	

		Amount of change	P				2.1	2.6		3.1	3.0	0.366
			A				0.8	3.7		3.5	4.0	

	Step 4	Measured value	P	33.7	3.6		35.5	7.1		35.3	10.1	
			A	33.7	4.1		36.4	4.7		37.7**	5.6	

		Amount of change	P				1.8	7.3		1.6	8.8	0.702
			A				2.6	5.1		4.0	3.4	

Group P: *n* = 14, group A: *n* = 11. Intra-group comparison at baseline *p<0.05 and **p<0.01 (1-sample *t* test). Inter-group comparison with group P (two-way-repeated measures of ANOVA)

**Table 6B:** Number of answers of subjects aged ≥55 years in cognitive tests

Item		Numerical item	Group	Baseline			Week 4			Week 8		*p* (Inter-group)
				Mean	SD		Mean	SD		Mean	SD	
Word memory test	Immediate recall	Measured value	P	4.5	0.7		4.8	0.9		4.6	0.9	
			A	4.6	0.9		5.0	0.9		4.8	0.7	

		Amount of change	P				0.3	1.0		0.1	1.1	0.906
			A				0.4	1.0		0.2	1.3	

	Immediate recall + cued recall	Measured value	P	5.7	0.8		5.5	1.2		5.9	0.8	
			A	5.9	1.0		5.9	1.0		6.1	0.7	

		Amount of change	P				–0.2	1.3		0.3	1.1	0.883
			A				–0.1	1.2		0.1	1.4	

	Recall after 5 min	Measured value	P	3.4	1.5		3.9	1.8		4.0	1.4	
			A	4.6	1.2		4.2	1.2		4.4	1.3	

		Amount of change	P				0.5	2.4		0.6	2.1	0.343
			A				–0.4	1.2		–0.2	1.9	

	Recall after 5 min + cued recall	Measured value	P	4.6	1.2		4.7	1.6		4.9	1.3	
			A	5.4	1.0		4.8	1.2		5.8	0.8	

		Amount of change	P				0.1	1.8		0.3	2.1	0.428
			A				–0.6	1.2		0.4	1.4	

Verbal fluency test	Names of vegetables	Measured value	P	15.7	3.2		15.1	5.1		16.2	3.5	
			A	16.1	3.6		16.1	3.5		17.3	4.4	

		Amount of change	P				–0.6	3.6		0.5	3.4	0.837
			A				0.0	3.1		1.2	3.0	

	Words that begin with “a”	Measured value	P	13.4	3.8		15.1*	5.1		14.3	3.3	
			A	13.5	4.1		13.6	3.8		15.5	3.7	

		Amount of change	P				1.7	2.2		0.9	3.2	0.198
			A				0.1	4.0		2.0	4.4	

	Animal words	Measured value	P	16.9	5.8		19.4	6.3		19.8	6.4	
			A	16.8	4.0		18.3	4.3		18.6*	4.3	

		Amount of change	P				2.5	5.8		2.8	5.0	0.756
			A				1.5	3.4		1.8	2.6	

Stroop test	Step 1	Measured value	P	58.1	6.2		57.6	7.3		59.5	8.7	
			A	56.3	6.3		59.5*	7.5		61.0**	6.2	

		Amount of change	P				–0.5	5.6		1.4	5.7	0.110
			A				3.2	4.9		4.7	4.1	

	Step 2	Measured value	P	47.8	6.0		49.6	4.5		50.6	7.8	
			A	48.1	6.2		51.1*	6.3		52.7**	5.3	

		Amount of change	P				1.8	4.7		2.8	7.0	0.619
			A				3.0	4.9		4.6	5.0	

	Step 3	Measured value	P	38.3	3.5		38.8	3.7		41.2**	4.2	
			A	38.8	3.7		41.5**	4.3		41.8**	4.3	

		Amount of change	P				0.5	3.1		2.8	3.2	0.160
			A				2.6	3.2		3.0	3.6	

	Step 4	Measured value	P	33.5	4.1		34.8	3.4		36	4.4	
			A	33.4	4.5		34.2	4.1		35.4	5.0	

		Amount of change	P				1.3	2.7		2.5	4.1	0.946
			A				0.8	5.2		1.9	5.6	

Group P: *n* = 12, group A: *n* = 17. Intra-group comparison at baseline *p<0.05 and **p<0.01 (1-sample *t* test). Inter-group comparison with group P (two-way-repeated measures of ANOVA).

## Abbreviations

BP: blood pressure
bpm: beats per minutes
HDS-R: physical tests and the Hasegawa Dementia Scale-Revised

## References

[1] Gong M, Bassi A (2016). Carotenoids from microalgae: a review of recent developments. Biotechnol Adv.

[2] Nakajima Y, Inokuchi Y, Shimazawa M, Otsubo K, Ishibashi T, Hara H (2008). Astaxanthin, a dietary carotenoid, protects retinal cells against oxidative stress *in-vitro* and in mice *in-vivo*. J Pharm Pharmacol.

[3] Nagaki A, Hayasaka S, Yamada T, Hayasaka Y, Sanada M, Uonomi T (2002). Effects of astaxanthin on accommodation, critical flicker fusion, and pattern visual evoked potential in visual display terminal workers. J Trad Med.

[4] Manabe E, Handa O, Naito Y (2008). Astaxanthin protects mesangial cells from hyperglycemia-induced oxidative signaling. J Cell Biochem.

[5] Aoi W, Naito Y, Takanami Y (2008). Astaxanthin improves muscle lipid metabolism in exercise via inhibitory effect of oxidative CPT I modification. Biochem Biophys Res Commun.

[6] Aoi W, Naito Y, Sakuma K (2003). Astaxanthin limits exercise-induced skeletal and cardiac muscle damage in mice. Antioxid Redox Signal.

[7] Tominaga K, Hongo N, Karato M, Yamashita E (2012). Cosmetic benefits of astaxanthin on humans subjects. Acta Biochim Pol.

[8] Kishimoto Y, Tani M, Uto-Kondo H (2010). Astaxanthin suppresses scavenger receptor expression and matrix metalloproteinase activity in macrophages. Eur J Nutr.

[9] Kuraji M, Matsuno T, Satoh T (2016). Astaxanthin affects oxidative stress and hyposalivation in aging mice. J Clin Biochem Nutr.

[10] Hussein G, Nakagawa T, Goto H (2007). Astaxanthin ameliorates features of metabolic syndrome in SHR/NDmcr-cp. Life Sci.

[11] Yamashita E (2015). Industrial production of astaxanthin using the green algae *Haematococcus pluvialis* and it use. Nippon Nogeikagaku Kaishi.

[12] Kitamura A, Hirai K, Yamashita E (2015). Commercial production of astaxanthin using green algae in the family *Haematococcus*. Seibutsu-kogaku Kaishi.

[13] Johnson EA, Lewis M (1979). Astaxanthin formation by the yeast *Phaffia rhodozyma*. J Gen Microbiol.

[14] Andrewes AG, Starr MP (1976). (*3R,3'R*)-astaxanthin from the yeast *Phaffia rhodozyma*. Phytochem.

[15] Andrewes AG, Phaffia HJ, Starr MP (1976). Carotenoids of *Phaffia rhodozyma*, a red-pigmented fermenting yeast. Phytochem.

[16] Tsubokura A, Yoneda H, Mizuta H (1999). *Paracoccus carotinifaciens* sp. nov., a new aerobic gram-negative astaxanthin-producing bacterium. Int J System Bacteriol.

[17] Katsumata T, Ishibashi T, Kyle D (2014). A sub-chronic toxicity evaluation of a natural astaxanthin-rich carotenoid extract of *Paracoccus carotinifaciens* in rats. Toxicol Rep.

[18] Maoka T, Yasui H, Ohmori A (2013). Anti-oxidative, anti-tumor-promoting, and anti-carcinogenic activities of adonirubin and adonixanthin. J Oleo Sci.

[19] Lim SY, Kim EJ, Kim A, Lee HJ, Choi HJ, Yang SJ (2016). Nutritional factors affecting mental health. Clin Nutr Res.

[20] Kaneai N, Sumitani K, Fukui K, Koike T, Takatsu H, Urano S (2016). Tocotrienol improves learning and memory deficit of aged rats. J Clin Biochem Nutr.

[21] Krikorian R, Shidler MD, Nash TA (2010). Blueberry supplementation improves memory in older adults. J Agric Supplement Chem.

[22] Santos RF, Galduróz JC, Barbieri A, Castiglioni ML, Ytaya LY, Bueno OF (2003). Cognitive performance, SPECT, and blood viscosity in elderly non-demented people using *Ginkgo biloba*. Pharmacopsychiatry.

[23] Katagiri M, Satoh A, Tsuji S, Shirasawa T (2012). Effects of astaxanthin-rich *Haematococcus pluvialis* extract on cognitive function: a randomised, double-blind, placebo-controlled study. J Clin Biochem Nutr.

[24] Nakagawa K, Kiko T, Miyazawa T (2011). Antioxidant effect of astaxanthin on phospholipid peroxidation in human erythrocytes. Br J Nutr.

[25] Fukuda M, Ishibashi T, Maoka T (2017). Analysis of adonixanthin and adonirubin in human Serum by ultra performance liquid chromatography (UPLC)-MS/MS. Carotenoid Sci.

[26] Imamura Y (2001). Manual for the Clinical Assessment of Higher Brain Function 2000, revised 2nd edition.

[27] Uemura K, Kanno T, Kato Y (2006). Simple and effective assessment of posttraumatic higher brain function disorders with special reference to the prefrontal area. Minimally Invasive Neurosurgery and Multidisciplinary Neurotraumatology.

[28] Clark DG, McLaughlin PM, Woo E (2016). Novel verbal fluency scores and structural brain imaging for prediction of cognitive outcome in mild cognitive impairment. Alzheimers Dement (Amst).

[29] Hakoda Y, Watanabe M (2004). New Stroop Test II.

[30] Nouchi R, Taki Y, Takeuchi H (2013). Brain training game boosts executive functions, working memory and processing speed in the young adults: a randomized controlled trial. PLoS One.

[31] Salthouse TA (2009). When does age-related cognitive decline begin?. Neurobiol Aging.

[32] Ikeda Y, Tsuji S, Satoh A, Ishikura M, Shirasawa T, Shimizu T (2008). Protective effects of astaxanthin on 6-hydroxydopamine-induced apoptosis in human neuroblastoma SH-SY5Y cells. J Neurochem.

[33] Kiko T, Nakagawa K, Satoh A (2012). Amyloid β levels in human red blood cells. PLoS One.

[34] Lee DH, Lee YJ, Kwon KH (2010). Neuroprotective effects of astaxanthin in oxygen-glucose deprivation in SH-SY5Y cells and global cerebral ischemia in rat. J Clin Biochem Nutr.

[35] Yook JS, Okamoto M, Rakwal R (2016). Astaxanthin supplementation enhances adult hippocampal neurogenesis and spatial memory in mice. Mol Nutr Food Res.

[36] Ohi Y, Kitamura A, Tsukahara H (2011). Pharmacokinetic of astaxanthin after oral administration of a soft gel capsule preparation. Rinshoiyaku.

[37] Saini RK, Nile SH, Park SW (2015). Carotenoids from fruits and vegetables: chemistry, analysis, occurrence, bioavailability and biological activities. Food Res Int.

[38] Qiu C, Winblad B, Fratiglioni L (2005). The age-dependent relation of blood pressure to cognitive function and dementia. Lancet Neurol.

[39] Inoue Y, Shimazawa M, Nagano R (2017). Astaxanthin analogs, adonixanthin and lycopene, activate Nrf2 to prevent light-induced photoreceptor degeneration. J Pharmacol Sci.

[40] Sandberg M, Patil J, D'Angelo B, Weber SG, Mallard C (2014). NRF2-regulation in brain health and disease: implication of cerebral inflammation. Neuropharmacology.

